# Climate Change Impact on Neotropical Social Wasps

**DOI:** 10.1371/journal.pone.0027004

**Published:** 2011-11-02

**Authors:** Alain Dejean, Régis Céréghino, James M. Carpenter, Bruno Corbara, Bruno Hérault, Vivien Rossi, Maurice Leponce, Jérome Orivel, Damien Bonal

**Affiliations:** 1 CNRS - UMR 8172, Écologie des Forêts de Guyane (Ecofog), Campus Agronomique, BP 709, Kourou, France; 2 Université de Toulouse; Université Paul Sabatier, Toulouse, France; 3 CNRS - UMR 5245, Laboratoire d'Ecologie Fonctionnelle et Environnement (EcoLab), Toulouse, France; 4 Division of Invertebrate Zoology, American Museum of Natural History, New York, New York, United States of America; 5 CNRS - UMR 6023, Laboratoire Microorganismes, Génome et Environnement, Université Blaise Pascal, Complexe Scientifique des Cézeaux, Aubière, France; 6 Clermont Université, Université Blaise Pascal, LMGE, BP 10448 F-63000, Clermont-Ferrand, France; 7 Université des Antilles et de la Guyane; Ècologie des Forêts de Guyane (UMR-UAG 43), Campus Agronomique, Kourou, France; 8 CIRAD; Écologie des Forêts de Guyane (UMR-CIRAD 93), Campus Agronomique, Kourou, France; 9 Biological Evaluation Section, Royal Belgian Institute of Natural Sciences, Brussels, Belgium; 10 INRA - Nancy, UMR EEF, Champenou, Nancy, France; University of Lausanne, Switzerland

## Abstract

Establishing a direct link between climate change and fluctuations in animal populations through long-term monitoring is difficult given the paucity of baseline data. We hypothesized that social wasps are sensitive to climatic variations, and thus studied the impact of ENSO events on social wasp populations in French Guiana. We noted that during the 2000 La Niña year there was a 77.1% decrease in their nest abundance along ca. 5 km of forest edges, and that 70.5% of the species were no longer present. Two simultaneous 13-year surveys (1997–2009) confirmed the decrease in social wasps during La Niña years (2000 and 2006), while an increase occurred during the 2009 El Niño year. A 30-year weather survey showed that these phenomena corresponded to particularly high levels of rainfall, and that temperature, humidity and global solar radiation were correlated with rainfall. Using the Self-Organizing Map algorithm, we show that heavy rainfall during an entire rainy season has a negative impact on social wasps. Strong contrasts in rainfall between the dry season and the short rainy season exacerbate this effect. Social wasp populations never recovered to their pre-2000 levels. This is probably because these conditions occurred over four years; heavy rainfall during the major rainy seasons during four other years also had a detrimental effect. On the contrary, low levels of rainfall during the major rainy season in 2009 spurred an increase in social wasp populations. We conclude that recent climatic changes have likely resulted in fewer social wasp colonies because they have lowered the wasps' resistance to parasitoids and pathogens. These results imply that Neotropical social wasps can be regarded as bio-indicators because they highlight the impact of climatic changes not yet perceptible in plants and other animals.

## Introduction

The impact of global warming has become obvious in high latitude regions where melting ice and softening tundra are causing profound changes. Species only survive if their niches are wide enough to overcome the changes related to these new climatic conditions. Many plants, for example, which are already adapted to the large temperature differences between hot summers and cold winters, bloom earlier, and, like sedentary animal species are responding at the population level by the slow, poleward shift of their ranges [1], [2]. However, niche conservatism with respect to climatic factors also exists, so that among closely-related species some are resistant to moving into novel climatic environments [3], [4].

In tropical forests, the major threats to biodiversity have mostly been attributed to deforestation and associated events such as fragmentation, habitat loss, fires and local climate change [5]. In fact, unspoiled tropical rainforests were formerly thought to be essentially unaffected by global warming due to lower increases in temperatures compared to forests at higher latitudes [6]. Nevertheless, the rate of warming in Amazonia was about 0.25°C per decade during the late 20^th^ century, and temperatures are projected to rise 3.3°C on average during the 21^st^ century due to mid-range greenhouse-gas emissions [5], [7]. This rise in temperature needs to be seen in light of the fact that tropical terrestrial ectotherms, including insects, have a narrower thermal tolerance than higher-latitude species, and are currently living very close to their maximum temperature limits [8]–[11]. Therefore, warming in the tropics is likely to have deleterious consequences as lowland biotas might be unable to tolerate even the smallest temperature change [12]–[14]. One example of an unexpected consequence can be seen for caterpillars that, when they are able to tolerate higher temperatures, develop before their parasitoid wasps are mature, decreasing the level of parasitism, and triggering a proliferation of the Lepidoptera [15].

Furthermore, there has been an increase in the frequency and intensity of El Niño Southern Oscillation (ENSO) events, most likely related to global warming, since 1976 [6], [16]–[19] (see definitions in Methods S1). Certain tropical regions, including Southeast Asia, New Guinea, northern Australia, West Africa, southern Mesoamerica, and central-eastern Amazonia have been affected by severe droughts associated with El Niño events [7], [20]. The negative impact of these droughts has been noted on above-ground forest biomass production [21] (Phillips *et al.*, 2009), plant survival and growth [22]–[24], vertebrates [25]–[27] and some insects [28], [29], while outbreaks of Lepidoptera and locusts have resulted in the defoliation of trees already stressed by drought [30], [31]. During La Niña events, which sometimes follow El Niño episodes, the climatic anomalies include increased cloud cover and rainfall. Their effect on plants and animals has received little attention because, in addition to having a lower frequency, the impact seems slight compared to the heavy droughts followed by forest fires that occur during El Niño episodes.

We were in the process of conducting a baseline study on the distribution and abundance of social wasps (Vespidae) nesting along forest edges in French Guiana [32], [33] when the 1997–1998 El Niño event was forecast. Recorded as the most severe and widespread in history, this event was followed by a 2-year La Niña episode [20], [34]. We hypothesized that vespid abundance and diversity might be strongly affected by the climatic fluctuations associated with this El Niño-La Niña event, because their life cycles are shaped by seasonality, causing populations to increase during the dry season [35]–[37] (but see [38] for tropical montane species). Furthermore, Vespidae are very sensitive to both drought [39]–[41] and increased humidity [36], [37], [42]–[44]. Consequently, we addressed the following questions. Were social wasp populations affected by these events? If so, which climatic factors contributed to these changes? Is the effect on social wasp populations temporary (in which case they quickly recover to their previous levels) or do these events change their abundance and/or distribution for a long time?

## Materials and Methods

### Ethics Statement

This study was conducted according to the European laws for scientific research currently in force. Sample collections necessary to scientific research were authorized by the French *Office National des Forêts* (*ONF*) (see *ONF-Guyane* at http://www.onf.fr/guyane/@@index.html).

### Site and the climate of French Guiana

This study was conducted in the Sinnamary district, French Guiana between 1997 and 2009 in natural areas free from any kind of known pollution. These areas are situated on both sides (up to 1 km deep) along the last 10 kilometres of the road leading to the Petit Saut dam (5° 03′ 45″ N; 53° 02′ 46″ W; hereafter ‘Petit Saut’).

French Guiana has an equatorial climate with four unequal periods: the “dry season” (July-November), the “short rainy season” (December-February), a decrease in precipitation levels during March, and the “major rainy season” (April-June). ENSO affects the Guianese climate by causing it to be drier during El Niño events, whereas increased levels of precipitation are related to La Niña episodes. Also, an intensification in the tropical Atlantic north-south sea surface temperature gradient can enhance the duration and intensity of the dry season in south-eastern Amazonia, including the Guiana Shield (see details in Methods S1).

### Collection of climatic data

To test the influence of climatic parameters on wasp populations, we used data from two weather stations situated ca. 25 km north-east of Petit Saut. A rain gauge installed at Petit Saut (5° 03′ 39″ N; 53° 02′ 36″ W) and monitored over 12 years showed only very slight variation with respect to these stations. We obtained the amounts of rainfall between 1980 and 1995 from a rain gauge situated at Pointe Combi (5° 16′ 28″ N; 52° 53′ 39″ W), and all climatic parameters including rainfall (mm/month), average monthly temperature (°C), average humidity (%), and global solar radiation (Joules/cm2/day) between 1996 and 2009 from the automatic weather station (Enerco405 AK, *Cimel Electronique*, Paris, France) at Paracou (5° 16′ 44″ N; 52° 55′ 31″ W).

### Surveys conducted in a plantation before and after the 1997–2000 ENSO event

We conducted a survey of the social wasp population found in a hillside plantation of grapefruit trees (*Citrus grandis*) situated 9 km from Petit Saut near St. Elie using data obtained in December 1992 [32] as a reference. Note that it is possible to census active nests fairly accurately, even in relatively dense forest. Indeed, it is unlikely that active colonies were overlooked because social wasp nests are easily detectable to a seasoned researcher. Moreover, the sedentary colonies are relatively large and the wasps, which generally have an aposematic coloring, are very active during the daytime.

No chemical treatments have ever been used on this experimental plantation, and weed and grasses were removed each year by hand. The same surveys were conducted in December 2006, 2007 and 2010 on the same 75 grapefruit trees in the plantation, 38 young *Astrocaryum sciophilum* palm trees situated in the same patch at the edges of the plantation, and along 800 m of forest edge lining the plantation (5 m in depth; ca. 1400 trees) (Table 1).

**Table 1:** Variation in the number of occupied wasp nests (all species pooled).

	Plantation*	Forest edge**	Palm trees*
Dec-1992	16/75	77	28/38
Dec-2006	7/75	2	2/38
Dec-2007	6/75	1	1/38
Dec-2010	7/75	1	1/38

* =  number of trees with wasp nests;

** =  number of wasp nests along the same perimeter. The data were obtained before and after the 1998–2000 ENSO event in the Saint Elie plantation and forest edges all around (Sinnamary district). Statistical comparisons. (1) Friedman *Chi*-square test  =  8.3333, df  =  3, P = 0.0396. (2) GLM Poisson model: the intercept corresponds to the effect ‘year *Dec 06*’ and the ‘trees of the forest edge’. The effect of grapefruit trees and *A. sciophilum* is significant as well as that of the year *Dec 92* at P<0.001. Tukey *post-hoc* test: the number of wasp nests recorded during the *Dec 92* survey is significantly different from that of the three other years (*Dec 06*, *Dec 07*, and *Dec 10*) at P<0.001; the differences between the number of wasp nests recorded during these three latter years were not significant. (3) Under the model: N_t_  =  a * N_t-1_ * ε, assuming a  =  1, the passage from 121 wasp nests in 1992 to 11 wasp nests in 2006 has a probability of 0.0355. It is therefore unlikely that this passage was due to demographic stochasticity only.

For statistical comparisons, using the *Friedman rank sum test*, we first tested if a difference in the number of wasp nests existed between the years compared, taking into account the pairwise nature of the comparison. The results allowed us to then reject the hypothesis that the numbers of wasp nests are identically distributed across the 4-year survey period. To identify what year(s) were responsible for the difference, we built a GLM Poisson model which explained the number of wasp nests by census year and tree type (i.e., palm trees, citrus trees and trees situated along the plantation edge). Indeed, because the numbers of wasp nests (Nw) is an account variable, it is likely that they are best modeled using a Poisson distribution with parameter λ: *Nw∼P(*λ*)*. To link Nw to the variables *years* and *trees*, we assume that λ is a function of these two variables: λ  =  exp (θ* *years* + *trees*). (3) Because the previous statistic does not exclude the possibility that the difference could be due to demographic stochasticity, we supposed that the number of wasp nests for the year t, N_t_, depends on the number of wasp nests for the year t_-1_, N_t-1_, so that: N_t_  =  a * N_t-1_ * ε, with ε being distributed according to a lognormal law, LN(0, σ). If the fluctuations observed are only due to demographic stochasticity with no long-term trend, then fitness “a” is equal to 1. By pooling all of the wasp nests regardless of tree species, we obtained 121, 11, 8, and 9 wasp nests in 1992, 2006, 2007 and 2010, respectively. From the 1992–2010 data, σ is estimated at (0.355). Using this estimation, we computed the probability that the number of wasp nests dropped to 11 in 2006 starting from 121 in 1992 under the assumption a  =  1.

### Surveys conducted along forest edges at the onset, during and after the 1997–2000 ENSO event

The same survey was replicated in December 1997, 2002 and 2007 (December is the month when wasp nests are the most abundant [37]. We located all of the wasp nests along ca. 5 km of forest edges lining small streams and the road that penetrates into the pristine forest at Petit Saut. We thoroughly inspected each plant taller than 0.75 m, as well as hollow logs and shelters formed by erosion (i.e., overhanging rocks or dirt). This corresponded to 15,235 plants inspected plus shelters other than plants (see also [33]). The wasps were identified to species and voucher specimens were deposited in the American Museum of Natural History, New York.

We conducted the same type of survey in six 400-m-long zones of forest edge lining the road leading to Petit Saut in December 1998, 2002 and 2006. The first sampling period began at the onset of the La Niña episode (just after the1997–1998 El Niño event) to determine its impact on the number of wasp populations. We did not try to identify the wasps to species.

Diversity statistics were calculated using EstimateS 7.5 software [45], with 500 randomizations of the sampling order without replacement. Statistical comparisons, including the Mann-Whitney test, Kruskal-Wallis test and Pearson's correlation coefficient, were conducted using Statistica 7.1 software (Statsoft; Tulsa, OK, USA). To bring out the relationships between climatic variables and wasps during the years 1997–2009, we used the Self-Organizing Map algorithm (SOM, neural network; [46]) presented in Methods S2.

### Long-term (13-year) studies

To determine exactly when climate change began to impact social wasp populations, we conducted two series of surveys each July between 1997 and 2009. The first study was conducted by thoroughly examining each tree or plant along 200 m of the stream situated at kilometre 23 (KP23) on the road leading to Petit Saut, an area with a particularly high density of social wasp nests. Here, too, we did not try to identify the wasps to species. The second study was inspired by the results obtained by Corbara et al. [33] showing that *Clusia grandiflora* trees very frequently shelter wasp nests under their wide, thick leaves. Thus, each year in July, we monitored *C. grandiflora* individuals growing along the road leading to the Petit Saut dam between KP20 and KP21. This study focused only on *Polybia bistriata*, a swarm-founding epiponine whose nests are protected by an envelope, because this species is by far the most frequent ([33]; Table S1), and because the only other four wasp species noted there were very poorly and irregularly represented.

Using a generalized linear model [47], we analyzed the variations in the number of wasp nests along the 200-m-long zone of the stream at KP23 and the percentages of *C. grandiflora* trees sheltering wasp nests between KP20 and KP21.

## Results

### Surveys conducted on a plantation before and after the 1997–2000 ENSO event

During the four series of studies conducted in the St. Elie plantation before and after the 1997-2000 ENSO event, we noted a significant decrease in the number of social wasp nests recorded between 1992 and 2006, 2007 and 2010 on the grapefruit trees, palm trees and forest edges lining the plantation, while the difference was not significant between 2006, 2007 and 2010 (Table 1).

### Surveys conducted along forest edges at the onset, during and after the 1997–2000 ENSO event

**Figure 1:**
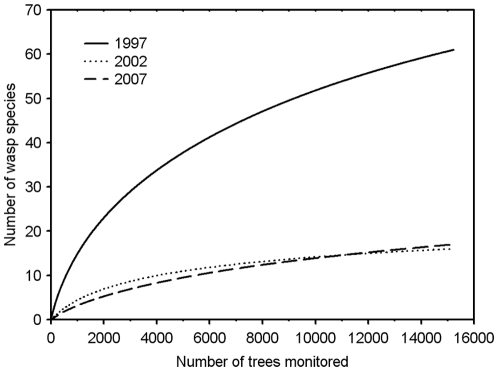
Species accumulation curves for the social wasps. Sampling was conducted in 1997, 2002 and 2007 along ca. 5 km of forest edges around Petit Saut. These surveys occurred before and after the 1997–2000 ENSO event. A total of 61 social wasp species was noted in 1997 *vs*. 16 in 2002 and 17 in 2007. Kruskal-Wallis test: H^3^_180_  =  82.15; P<0.0001; Dunn's multiple comparison test: 1997 *vs.* 2002 and 1997 *vs*. 2007: P<0.001; 2002 *vs*. 2007: NS (details in Table S1).

We noted a significant decrease in social wasp populations between 1997, 2002 and 2007 (424 wasp nests in 1997 *versus* 97 in 2002, or a decrease of 77.1% in the number of wasp nests, and only 67 in 2007) along the ca. 5 km of forest edges. The difference between 2002 and 2007 was not significant (Table S1). Diversity followed the same pattern with 61 species recorded in 1997 *versus* 16 and 17 thereafter (Fig. 1); 70.5% of the species were never observed again.

Similar results were noted between December 1998, 2002 and 2006 for the six sites selected along the road leading to Petit Saut. By excluding the influence of the El Niño episode that preceded the year 1998, these surveys show that the decrease in social wasp populations occurred during the La Niña that followed (here, all of the differences were significant; Fig. S1).

### Long-term (13-year) studies

**Figure 2:**
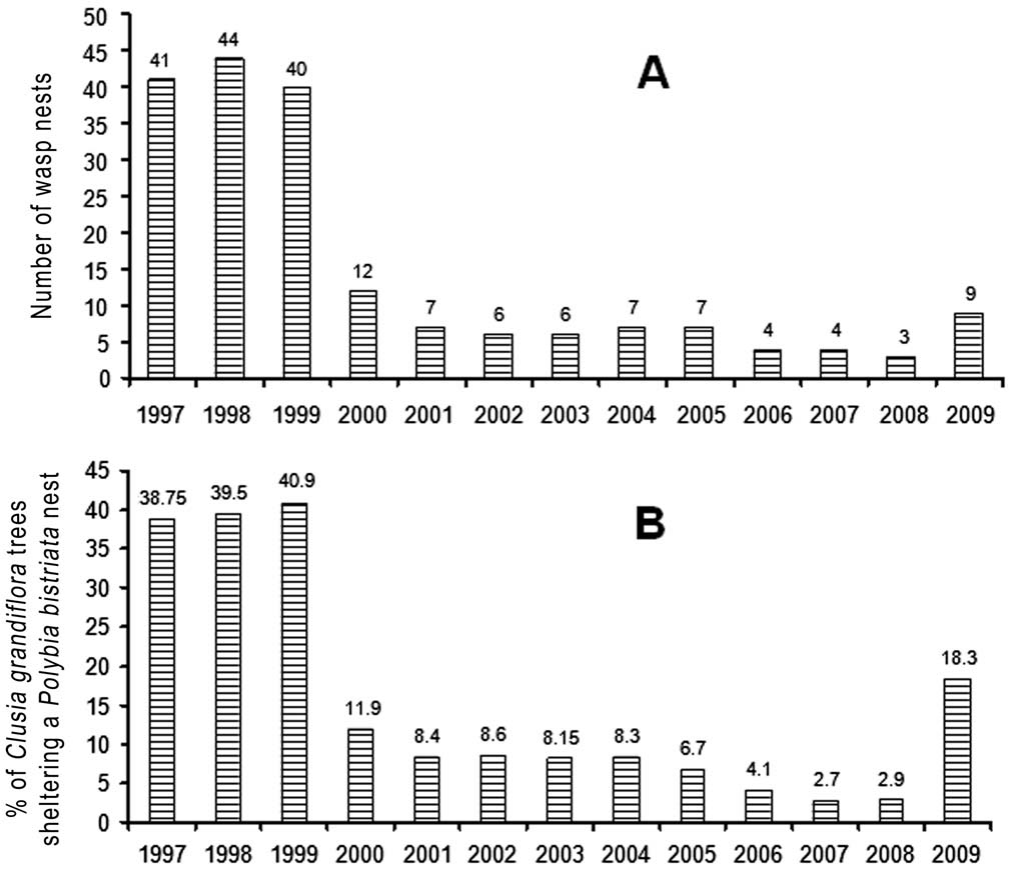
Variation in the number of wasp nests during a 13-year-long survey (1997–2009; wasp nests monitored each July). A. Along 200 m of the stream situated at kilometre 23 on the road leading to the Petit Saut dam, all species pooled. The number of wasp nests was significantly higher between 1997 and 1999 than between 2000 and 2008 (Mann-Whitney test: U = 0.000: P<0.01). B. Variation in the percentages of *Clusia grandiflora* trees sheltering a *Polybia bistriata* nest between 1997 and 2009 (120 to 149 trees monitored each year in July). GLIM statistic; the complete model calculated from the core data resulted in a significant difference (GLIM; 13-year study; χ^2^_12_  =  203; P<10^−5^), while the simplified model (four groups of years: 1997-1999; 2000-2005; 2006-2008; 2009) was not statistically different from the complete model (χ^2^_3_  =  195.8, P<10^−5^; Δχ^2^_9_  =  7.19, P = 0.104). This permitted us to conduct pairwise comparisons showing that these four groups of years were significantly different from each other (P = 0.034).

**Figure 3:**
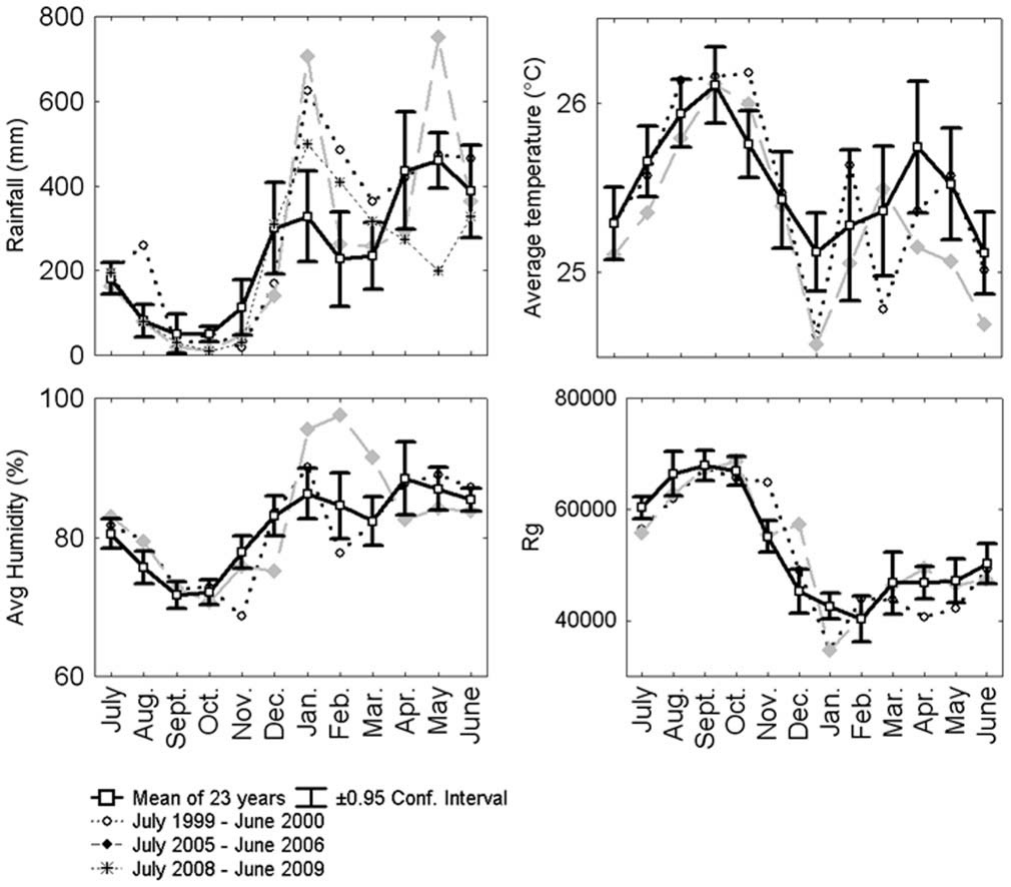
Monthly rainfall (30-year period prior to July 2009), average temperature, relative humidity, and global solar radiation (14-year period). Special reference for three years: the July 1999-June 2000 period represented by dotted lines; the July 2005-June 2006 period represented by a segmented line, and for rainfall only; and the July 2008-June 2009 period represented by a smaller dotted line. Note that humidity, temperature and solar radiation were correlated with rainfall (rainfall *vs*. humidity, r = 0.74, P<0.001; rainfall *vs*. temperature:  = −0.48, P<0.001; rainfall *vs*. solar radiation, r = −0.70, P<0.001). The tendency curves show a decrease in rainfall during the dry season (Y  =  −5.35X + 626.4) and an increase during both only the short rainy season (Y  =  +11.98X + 705.5) and the entire rainy season (Y  =  +10.22X + 2228.4).

During the 13-year-long studies, we noted similar patterns of variation in abundance for all wasp species pooled and *P. bistriata* alone (Fig. 2). The latter study permitted us to statistically differentiate four periods: ca. 40% of the *C. grandiflora* plants sheltered a wasp nest from 1997 to 1999, 8.7% from 2000 to 2005, 3.3% from 2006 to 2008, and 18.3% in 2009 (Fig. 2B). The major decrease in the number of social wasp nests during the 2000 La Niña year was therefore confirmed, while it highlighted that further ENSO events also played a role. Indeed, a second major decrease in social wasp populations occurred in 2006, and corresponded to another La Niña year, with large climatic contrasts between a severe dry season followed by particularly heavy rainfall during the short rainy season (Figs. 3 and S5). On the contrary, the increase in social wasp populations noted during the year 2009 corresponded to low rainfall during the major rainy season in an El Niño event (Figs. 3 and S5).

### Relationships between social wasp populations and climatic variables

**Figure 4:**
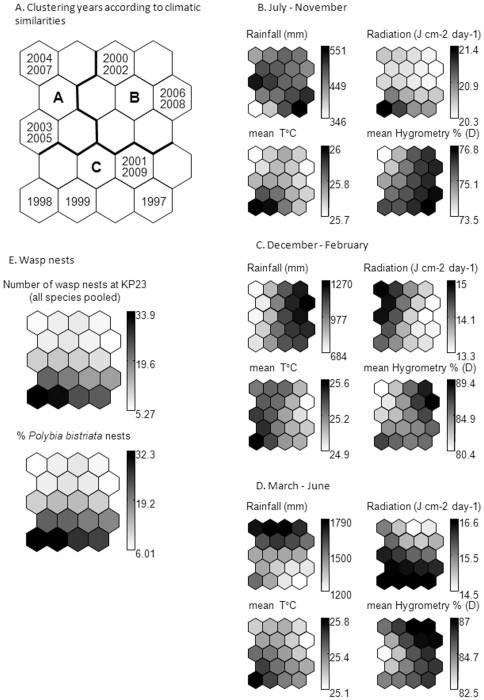
Distribution and clustering of the years 1997–2009 on the Self-Organising Map (SOM). A- Distribution according to the different climatic parameters that characterize them (small maps); clusters A-C represent subsets of years with similar climatic conditions according to the 12 input variables derived from the k-means algorithm applied to the weights of these variables in the 20 output neurons of the SOM. B-D- Gradient analysis of the climatic parameters that permitted us to establish the classification (dark  =  high value, light  =  low value). Each small map representing one variable can be compared to (or superimposed on) the map representing the distribution of years presented in Fig. 3A. E- Mean value for the number of wasp nests recorded at KP23 and percentages of *Polybia bistriata* nests recorded each year in July calculated in each output neuron of the SOM previously trained with climatic parameters. Dark represents a high value, while light is low.

Fluctuations in social wasp populations and climatic variables showed congruent patterns (Fig. 4). Three clusters were delimited on the SOM according to the climatic parameters that characterize them (Fig. 4A) with solar radiation, temperature and humidity being correlated with rainfall (Fig. 3). Rather low rainfall during the short rainy season (December-February) followed by heavy rainfall during the major rainy season (March-June) contributed to the formation of cluster A for the years 2003, 2004, 2005 and 2007. Cluster B resulted from heavy rainfall during the entire rainy season (December-June) in 2000, 2002, 2006 and 2008, and cluster C from relatively low rainfall during either the short or the major rainy season during the years 1997, 1998, 1999, 2001 and 2009 (Fig. 4A). The year 2001 also belongs to this cluster even though the wasp population was low (Fig. 2).

The superimposition of the number of wasp nests at KP23 and the percentages of *C. grandiflora* sheltering a *P. bistriata* nest onto the framework built from the climatic parameters reveals a similar gradient for biological variables (Fig. 4E), suggesting that the climatic conditions during the 2000 La Niña year triggered the very strong decline in wasp populations noted in Fig. 2. These populations were unable to recover, likely because similar conditions occurred again in 2002, 2006 and 2008 (cluster B; Fig. 4A; both 2006 and 2008 correspond to la Niña episodes, see Fig. S2). The years 2003, 2004, 2005 and 2007 (cluster A; Fig. 4A) were also unfavourable to social wasps, this time due to particularly heavy rainfall during the major rainy season.

## Discussion

Due to similar abundance along forest edges between 1992 and 1999 [32], [33], social wasp populations nesting along forest edges in French Guiana were stable for a long time. In fact, before 1999, the number of lowland Neotropical social wasp nests decreased each year at the onset of the rainy season, and then returned to its initial level during the dry season, after swarming, as is known for other Neotropical areas [35]–[37]; the contrary is true for very dry areas [41]. Therefore, a major decrease in social wasp populations occurred during a particularly long La Niña episode and never recovered the pre-2000 level. This was confirmed by further observations (July and December 2010; in July 2011), with even a decrease in the number of social wasp nests noted at KP23, and between KP20 and KP21 (areas corresponding to Fig. 2). In spite of this strong decrease following the year 2000, we show that fluctuations in the number of social wasp nests occurred during the other La Niña and El Niño episodes and more generally after periods of heavy rainfall (Figs. 2 and 4).

The extreme contrasts in rainfall between the dry season and the onset of the short rainy season, noted in 1999–2000 and then again in 2005–2006 (Fig. 3), are likely responsible for much of the decrease in the number of wasp nests and the loss of diversity ([37]; this study). These results are alarming because this contrast increased during the study period (Fig. 3; Fig. S2) and is predicted to increase by 7% during the 21^st^ century in north-eastern Amazonia and French Guiana (in addition to a greater mean temperature) [6]–[8], [48]. Note that temperatures have increased by 1.6°C in French Guiana over the last 50 years [14]. Therefore, if social wasps live close to their maximum temperature limits in the tropics, as has generally been reported for ectotherms [10]–[13], they may be unable to withstand even small modifications to their environment or to resist pathogens.

In French Guiana certain social wasp species, including *P. bistriata*, adapt to seasonal variations by moving twice a year, installing their nests in shady areas close to the ground during the warm, dry seasons, and then moving to higher, better ventilated zones of the vegetation with the onset of the rainy season [37]. This illustrates the importance of temperature and humidity, both correlated with rainfall (Fig. 3), in their nesting habits. More generally, heavy rainfall during the short and/or the major rainy season(s) seems detrimental to social wasps (Fig. 4), while a slight increase in social wasp populations was noted after a far below average amount of rainfall during the major rainy season in 2009 (see Figs. 2 and 3).

Fluctuations in animal populations related to the prior conjunction of several abnormal climatic conditions have been noted for birds [27] and amphibians [49]–[51]. In birds, these parameters were related to food supply [52], while the extinction of amphibian populations likely occurred after consecutive years of unusually warm weather favouring the development of a fungal pathogen [51], [53], [54]. Also, Harvell et al. [62] suggested that global warming alters precipitation and humidity levels by facilitating ENSO events, something often associated with the greater presence of pathogens. They added that correlations between pathogens, their hosts and climate change are difficult to establish due to a lack of baseline data, the multivariate nature of climate change, and non-linear thresholds in both pathogens and in climatic processes.

It is unlikely that social wasp abundance decreased due to an increase in predator populations, especially army ants, which are considered the main predators of Neotropical social wasps [55]–[59]. Our presence in the field each week enabled us to note a decrease in the number of raids by army ants that forage in the vegetation. Furthermore, due to the abiotic conditions, these army ants avoid foraging along forest edges, the focal sites of this study [9], [33]. We therefore argue that two phenomena have likely combined to decrease the number of social wasp populations. First, that the decrease in social wasp populations occurred simultaneously in multiple species suggests the existence of a climate-related threshold (see also [26] for vertebrates). It is likely that most wasp colonies did not survive when rainfall and humidity increased in 1999–2000 and 2005–2006 in French Guiana (Figs. 2 and 4). In both cases, the envelope protecting the nests of most of the epiponine species was so soaked during the onset of the short rainy season that the combs situated at the base of large nests came loose and dropped off with a part of the brood. Furthermore, the workers continued to hunt, storing several hundred prey in any available space in their nest. These prey decayed in the humid nests, forcing the wasps to abscond several times, thus losing their nest and brood [37]. Second, the vulnerability of social wasps to changes in several climatic parameters might be related to a lower resistance to parasitoids and pathogens (see [54]). Indeed, even wasp species whose nests are devoid of an envelope (i.e., the Polistini, Mischocyttarini and *Apoica* among the Epiponini) suffered great losses, although they do not stock prey that can then decay. Also, during the year 2001, social wasp populations did not increase even though the climatic conditions were favourable. These observations suggest that limiting factors (likely pathogens) reached high levels in 2000 and continued into 2001, keeping social wasp population levels low. It is known, for instance, that Neotropical wasps are sensitive to gregarines (Apicomplexa) when air humidity increases [43], [44] (see also [60] for monarch butterflies). Sequencing Guianese wasp extracts during a La Niña year allowed DNA amplification from several potential pathogens, but the presence of each of them was noted in only one or a few samples, which is not consistent with an epidemic [37].

Parasitoid flies might also play a role as we noted their presence during the particularly wet 2006 rainy season, and observed females spending most of the daylight hours trying to lay eggs on wasp nests devoid of an envelope. Jeanne (pers. comm.) also noted a relationship between wet periods and phorid flies parasitizing wasp nests during a study in Venezuela on the protective role of the envelope of social wasp nests (see also [61]).

Therefore, social wasps can be affected by different enemies (i.e., parasitoids, apicomplexa, bacteria and viruses) acting simultaneously, and aided by higher temperatures [62] and/or greater humidity (this study), regardless of whether or not their nests are covered by an envelope. This has been noted for honey bees devastated by a co-infection by an iridescent virus and microsporidia in addition to infestations by *Varroa* mites [63].

In conclusion, that social wasp populations never recovered their pre-2000 levels in French Guiana indicates that major climatic changes probably occurred and are likely related to higher temperatures in conjunction with periods of heavy rainfall, particularly during the onset of the short rainy season (resulting in greater contrasts with the preceding dry season). Because climatologists predict that this situation will intensify, it might be useful to regard social wasps as bio-indicators highlighting changes not yet perceptible in plants and other animals.

## Supporting Information

Methods S1 **El Niño Southern Oscillation (ENSO) and the climate of French Guiana.**
(DOC)

Methods S2 **The Self-Organizing Map algorithm (SOM).**
(DOC)

Figure S1 **Variations in the number of wasp nests at the onset and after the 1998-2000 La Niña event.**
(DOC)

Figure S2 **Pluviometry in the area studied during the dry season, the short and the major rainy** seasons between 1980 and 2009.
(DOC)

Table S1 **Wasp species sampled during the surveys.**
(DOC)

## References

[1] Parmesan C, Yohe G (2003). A globally coherent fingerprint of climate change impacts across natural systems.. Nature.

[2] Root TL, Price J, Hall K, Schneider S, Rosenzweig C (2003). Fingerprints of global warming on wild animals and plants.. Nature.

[3] Wiens JJ, Graham CH (2005). Niche conservatism: integrating evolution, ecology and conservation biology.. Annu Rev Ecol Evol Syst.

[4] Parmesan C (2006). Ecological and evolutionary response to recent climate change.. Annu Rev Ecol Evol Syst.

[5] Malhi Y, Roberts JT, Betts RA, Killeen TJ, Li W (2008). Climate change, deforestation, and the fate of the Amazon.. Science.

[6] IPCC (2007). Climate Change 2007: the physical science basis..

[7] Malhi Y, Wright J (2004). Spatial patterns and recent trends in the climate of tropical rainforest regions.. Phil Trans R Soc London B.

[8] Heinrich B (1996). The thermal warriors: strategies of insect survival..

[9] Meisel JE (2006). Thermal ecology of the Neotropical army ant *Eciton burchelli*i.. Ecol Appl.

[10] Deutsch C, Tewksbury JJ, Huey RB, Sheldon K, Ghalambor C (2008). Impacts of climate warming on terrestrial ectotherms across latitude.. Proc Natl Acad Sc USA.

[11] Tewksbury JJ, Huey RB, Deutsch CA (2008). Putting the heat on tropical animals.. Science.

[12] Cowling SA, Betts RA, Cox PM, Ettwein VJ, Jones CD (2004). Contrasting simulated past and future responses of the Amazonian forest to atmospheric change.. Phil Trans R Soc London, B.

[13] Colwell RK, Brehem G, Cardelús CL, Gilman AC, Longino JT (2008). Global warming, elevation range shifts, and lowland biotic attrition in the wet tropics.. Science.

[14] Fonty E, Sarthou C, Larpini D, Ponge J-F (2009). A 10-year decrease in plant species richness on a neotropical inselberg: detrimental effects of global warning?. Glob Change Biol.

[15] Parks D (2011). Climate change and caterpillars.. http://www.earthwatch.org/europe/expeditions/exped_research_focus/rf-cluttonbrock-010420.

[16] Collins M (2005). El Niño- or La Niña-like climate change?. Climate Dyn.

[17] Tsonis AA, Elsner JB, Hunt AG, Jagger TH (2005). Unfolding the relation between global temperature and ENSO.. Geophys Res Let.

[18] Gergis JL, Fowler AM (2006). How unusual was late 20th century El Niño-Southern Oscillation (ENSO)? Assessing evidence from tree-ring, coral, ice-core and documentary palaeoarchives, A.D. 1525–2002.. Adv Geosc.

[19] Vargas G, Pantoja S, Rutlant JA, Lange CB, Ortlieb L (2007). Enhancement of coastal upwelling and interdecadal ENSO-like variability in the Peru-Chile Current since late 19th century.. Geophys Res Let.

[20] Kayano MT, Andreoli RV (2006). Relationships between rainfall anomalies over northeastern Brazil and the El Niño-Southern Oscillation.. J Geophysic Res.

[21] Phillips OL, Aragão L, Fisher JB, Lewis SM, Lloyd J (2009). Drought sensitivity of the Amazon rainforest.. Science.

[22] Harrison RD (2001). Drought and the consequences of El Niño in Borneo: a case study of figs.. Pop Ecol.

[23] Aiba S-I, Kitayama K (2002). Effects of the 1997-98 El Niño drought on rain forests of Mount Kinabalu, Borneo.. J Trop Ecol.

[24] Pau S, Okin GS, Gillespie TW (2010). Asynchronous response of tropical forest leaf phenology to seasonal and El Niño-driven drought.. PLoS ONE.

[25] Jaksic FM, Silva SI, Meserve PL, Gutierrez JR (1997). A long-term study of vertebrate predator responses to an El Niño (ENSO) disturbance in western South America.. Oikos.

[26] Pounds JA, Fogden MPL, Campbell JH (1999). Biological response to climate change on a tropical mountain.. Nature.

[27] Grant PR, Grant BR, Keller LF, Petren K (2000). Effects of El Niño events on Darwin's finch productivity.. Ecology.

[28] Harrison RD (2000). Repercussions of El Niño: drought causes extinction and the breakdown of mutualism in Borneo.. Proc R Soc London, B.

[29] Hoffmann AA, Hallas RJ, Dean JA, Schiffer M (2003). Low potential for climatic stress adaptation in a rainforest *Drosophila* species.. Science.

[30] Todd MC, Washington R, Cheke RA, Kniveton D (2002). Brown locust outbreaks and climate variability in southern Africa.. J Appl Ecol.

[31] Van Bael SA, Aiello A, Valderama A, Medianero H, Samaniego M (2004). General herbivore outbreak following an El Niño-related drought in a lowland Panamanian forest.. J Trop Ecol.

[32] Dejean A, Carpenter JM, Corbara B (1998). Nesting site selection by wasps in the Guianese rain forest.. Ins Soc.

[33] Corbara B, Carpenter JM, Céréghino R, Leponce M, Gibernau M (2009). Diversity and nest site selection of social wasps along Guianese forest edges: assessing the influence of arboreal ants.. C R Biol.

[34] NOAA (2010). http://www.cpc.ncep.noaa.gov/products/analysis_monitoring/lanina/enso_evolution-status-fcsts-web.pdf.

[35] Diniz IR, Kitayama K (1998). Seasonality of vespid species (Hymenoptera: Vespidae) in a central Brazilian cerrado.. Rev Biol Trop.

[36] Hunt JH, Brodie RJ, Carithers TP, Goldstein PZ, Janzen DH (2006). Dry season migration by costa rican lowland paper wasps to high elevation cold dormancy sites.. Biotropica.

[37] Dejean A, Carpenter JM, Gibernau M, Leponce M, Corbara B (2010). Nest relocation and high mortality rate in a Neotropical social wasp: impact of an exceptionally rainy La Niña year.. C R Biol.

[38] O'Donnell S, Joyce FJ (2001). Seasonality and colony composition in a montane tropical eusocial wasp.. Biotropica.

[39] Jeanne RL, Nordheim EV (1996). Productivity in a social wasp: per capita output increases with swarm size.. Behav Ecol.

[40] Santos GM de M, Bichara Filho CC, Resende JJ, da Cruz JD, Marques OM (2007). Diversity and community structure of social wasps (Hymenoptera: Vespidae) in three ecosystems in Itaparica island, Bahia state, Brazil.. Neotrop Entomol.

[41] Santos GM de M, Bispo PC, Aguiar CML (2009). Fluctuations in richness and abundance of social wasps during the dry and wet seasons in three phyto-physiognomies at the tropical dry forest of Brazil.. Environ Entomol.

[42] Hunt JH, O'Donnell S, Chernoff N, Brownie C (2001). Observations on two Neotropical swarm-founding wasps, *Agelaia yepocapa* and *A. panamaensis* (Hymenoptera: Vespidae).. Ann Entomol Soc Amer.

[43] Howard KJ, Jeanne RL (2004). Rates of brood development in a social wasp: effects of colony size and parasite infection.. Ins Soc.

[44] Bouwma AM, Howard K, Jeanne RL (2005). Parasitism in a social wasp: effect of gregarines on foraging behavior, colony productivity, and adult mortality.. Behav Ecol Sociobiol.

[45] Colwell RK (2005). http://purl.oclc.org/estimates.

[46] Kohonen T (2001). Self-Organizing Maps, 3^rd^ edn..

[47] Payne CD (1986). The generalised linear interactive modelling system.. Release 3.77.

[48] Li W, Fu R, Dickinson RE (2006). Rainfall and its seasonality over the Amazon in the 21st century as assessed by the coupled models for the IPCC AR4.. J Geophysic Res.

[49] Pounds JA, Bustamante MR, Coloma LA, Consuegra JA, Fogden MPL (2006). Widespread amphibian extinctions from epidemic disease driven by global warming.. Nature.

[50] Alford A, Bradfield KS, Richards SJ (2007). Global warming and amphibian losses.. Nature.

[51] Laurence WF (2008). Global warming and amphibian extinctions in eastern Australia.. Aust Ecol.

[52] Gaston AJ, Martin JL, Allombert S (2006). http://www.ace-eco.org/vol/iss/art4/.

[53] Smith KG, Lips KR, Chase JM (2009). Selecting for extinction: non random disease-associated extinction homogenizes amphibian biotas.. Ecol Let.

[54] Rohr JR, Raffel TR (2010). Linking global climate and temperature variability to widespread amphibian declines putatively caused by disease.. Proc Natl Acad Sc USA.

[55] Jeanne RL (1975). The adaptiveness of social wasp nest architecture.. Q Rev Biol.

[56] O'Donnell S, Jeanne RL (1990). Notes on an army ant (*Eciton burchellii*) raid on a social wasp colony (*Agelaia yepocapa*) in Costa Rica.. J Trop Ecol.

[57] Gotwald WH (1995). Army ants: the biology of social predation..

[58] Bouwma AM, Howard KJ, Jeanne RL (2007). Rates of predation by scouting-and-recruiting ants on the brood of a swarm-founding wasp in Costa Rica.. Biotropica.

[59] Kumar A, Longino JT, Colwell RK, O'Donnell S (2009). Elevational patterns of diversity and abundance of eusocial paper wasps (Vespidae) in Costa Rica.. Biotropica.

[60] Altizer SM, Oberhauser KS, Brower LP (2000). Associations between host migration and the prevalence of a protozoan parasite in natural populations of adult monarch butterflies.. Ecol Entomol.

[61] London KB, Jeanne RL (1999). Envelopes protect social wasps'nests from phorid infestation (Hymenoptera: Vespidae; Diptera: Phoridae).. J Kansas Entomol Soc.

[62] Harvell CD, Mitchell CE, Ward JR, Altizer S, Dobson AP (2002). Climate warming and disease risks for terrestrial and marine biota.. Science.

[63] Bromenshenk JJ, Henderson CB, Wick CH, Stanford MF, Zulich AW (2010). Iridovirus and microsporidian linked to honey bee colony decline.. PLoS ONE.

