# Supplementary material for: Climate Change Impact on Neotropical Social Wasps
Source: PLoS One. 2011 Nov 2;6(11):e27004. doi: 10.1371/journal.pone.0027004 (PMC3206903; doi:10.1371/journal.pone.0027004)
Supplement: Methods S1 — El Niño Southern Oscillation (ENSO) and the climate of French Guiana. (DOC) [file pone.0027004.s001.doc]

**Supplementary Methods S1**

**El Niño Southern Oscillation (ENSO) and the climate of French Guiana**

**ENSO**

Due to the rotation of the Earth, [trade winds](http://ww2010.atmos.uiuc.edu/(Gh)/wwhlpr/hurricane_globalwinds.rxml?hret=/guides/mtr/eln/def.rxml) in the tropical Pacific Ocean generally drive the surface waters westward where they become warmer due to longer exposure to solar heating. The El Niño Southern Oscillation (ENSO) is observed annually around Christmastime (‘El Niño’ means ‘the infant Jesus’ in Spanish), and lasts only a few weeks until the trade winds weaken, allowing the warmer waters of the western Pacific to flow eastward and reach the coast of Ecuador and Peru. Every 2 to 7 years, this situation can persist, giving rise to an “El Niño event”, or a warm period. La Niña, or a cold period, is essentially the opposite with stronger than usual trade winds that push the warm surface water away, so that the cold water in the eastern part of the tropical Pacific Ocean rises from below. El Niño and La Niña “episodes” are officially defined as sustained sea-[surface temperature](http://en.wikipedia.org/wiki/Surface_temperature) (SST) anomalies of a [magnitude](http://en.wikipedia.org/wiki/Magnitude_(mathematics)) greater than 0.5°C across the central tropical Pacific Ocean persisting for 5 months or more (strong ENSO events correspond to an SST of 3-4 degrees above average). El Niños can be followed by La Niña episodes, but La Niñas may begin on their own. In both situations, the resulting SST modifications impact atmospheric circulation patterns worldwide, causing a range of environmental changes with opposing effects (see Philander [1]).

**The climate of French Guiana**

Situated between 2°N and 6°N, French Guiana has an equatorial climate with constant winds and temperatures throughout the year. The amount of rainfall is related to the movements of the Atlantic intertropical convergence zone (ITCZ). The ITCZ is a belt of low pressure and converging trade winds that encircles the Earth near the Equator. It is formed by the vertical ascent of warm, moist air from the latitudes where the sun's rays are the most direct, so that it shifts position over the course of the year as it follows the sun's rays. Also, as a result of the asymmetry of continental geometry and air-sea interactions, the ITCZ shifts north of the Equator in the Atlantic [2,3]. During its oscillations, the ITCZ reaches Guianese coastal areas twice each year, delineating a seasonal cycle with four unequal periods. During the “dry season”, from July to November, the ITCZ lies north of French Guiana. As it moves southward the ITCZ is over French Guiana from December to February, corresponding to the “short rainy season”. When it reaches its extreme position during March, precipitation levels decrease; this is known as the “short dry season”. Then the ITCZ slowly moves northward and a new period of heavy rains occurs from April to June, corresponding to the “major rainy season” [4].

The northeastern Amazon and French Guiana are affected by ENSO due to a chain of correlated events between (1) sea surface temperatures (SSTs) in the equatorial Pacific, (2) wind stress in the western equatorial Atlantic and (3) equatorial Atlantic SSTs and sea surface height [5,6]. Like northeastern Amazonia, the Guianese climate is drier during El Niño events, while La Niña episodes are related to increased precipitation [7,8]. Also, the intensification of the tropical Atlantic north-south SST gradient can shift the ITCZ northwards, enhancing the duration and intensity of the dry season in much of southern and eastern Amazonia, including the Guiana Shield, as occurred in 2005 [9-11]. This resulted in a drought during the 2005 dry season (the driest recorded since 1980).

**References**

[1] Philander SGH (1990) El Niño, La Niña and the Southern Oscillation. Academic Press, San Diego.

[2] Hastenrath S, Lamb P (1978) On the dynamics and climatology of surface flow over the equatorial oceans. Tellus 30**:** 436-448.

[3] Grodsky SA, Carton JA (2003) The intertropical convergence zone in the South Atlantic and the equatorial cold tongue. J Climate 16: 723-733.

[4] Météo-France (2005) [www.meteo.fr/temps/domtom/antilles/pack-public/meteoPLUS/climat/clim_guy.htm](http://www.meteo.fr/temps/domtom/antilles/pack-public/meteoPLUS/climat/clim_guy.htm)

[5] Münnich M, Neelin JD (2005) Seasonal influence of ENSO on the Atlantic ITCZ and equatorial South America. Geophysic Res Let 32: L21709, doi: 10.1029/ 2005GL023900.

[6] Kayano MT, Andreoli RV (2006) Relationships between rainfall anomalies over northeastern Brazil and the El Niño-Southern Oscillation. J Geophysic Res 111**:** D13101, doi:10.1029/2005JD006142.

[7] Marengo JA, Nobre CA, Sampaio G (1998) On the associations between hydrometrical conditions in Amazonia and the extremes of the Southern Oscillation. J Climate 17: 2261-2280.

[8] Schöngart J, Junk WJ, Piedade MTF, Ayres JM, Huttermann A, Worbes M (2004) Teleconnection between tree growth in the Amazonian floodplains and the El Nino Southern Oscillation effect. Glob Change Biol 10: 683-692.

[9] Malhi Y, Roberts JT, Betts RA, Killeen TJ, Li W, Nobre CA (2008) Climate change, deforestation, and the fate of the Amazon. Science 319: 169-172.

[10] Marengo JA, Nobre CA, Tomasella J, Cardoso MF, Oyama DM (2008) Hydro-climatic and ecological behaviour of the drought of Amazonia in 2005. Phil Trans R Soc London B 363: 1773-1778.

[11] Lewis SL, Brando PM, Phillips OL, van der Heijden GMF, Nepstad D (2011) The 2010 Amazon drought. Science 331: 554-555.
