# Supplementary material for: Climate Change Impact on Neotropical Social Wasps
Source: PLoS One. 2011 Nov 2;6(11):e27004. doi: 10.1371/journal.pone.0027004 (PMC3206903; doi:10.1371/journal.pone.0027004)
Supplement: Methods S2 — The Self-Organizing Map algorithm (SOM). (DOC) [file pone.0027004.s002.doc]

**Supplementary Methods S2**. **The Self-Organizing Map algorithm (SOM)**. The SOM was used as a tool to establish congruent patterns between climatic data and biological variables characterizing social wasps.

To bring out the relationships between the years 1997-2009, climatic variables, and wasps, we used the Self-Organizing Map algorithm (SOM, neural network). Combining ordination and gradient analysis functions, the SOM is a convenient way to show high-dimensional ecological data in a readily interpretable manner without prior transformation. The SOM algorithm is an unsupervised learning procedure that transforms multi-dimensional input data into a two-dimensional map subject to a topological (neighbourhood preserving) constraint (details presented in Kohonen [1]). The SOM thus plots the similarities of the data by grouping similar data items together onto a 2D-space (displayed as a grid) using an iterative learning process that was presented in Park *et al.* [2]. The SOM algorithm is especially relevant for analyzing sets of variables that vary and co-vary in a non-linear fashion, and/or that have skewed distributions. Additionally, the SOM algorithm averages the input dataset using weight vectors through the learning process and thus removes noise. A full description of the modeling procedure employed here (i.e., training, map size selection, number of iterations, map quality values) was provided in Park *et al*. [2] and Céréghino and Park [3].

First, we wanted to classify years according to climatic conditions taking into consideration global solar radiation, monthly rainfall, average temperature and relative humidity between July 1996 and July 2009. The structure of the SOM for this analysis consisted of two layers of neurons connected by weights (or connection intensities): the input layer was composed of 12 neurons (4 climatic variables x 3 periods) connected to the 13 consecutive years (1997-2009), and the output layer was composed of 20 neurons displayed as hexagonal cells organized on a grid consisting of 4 rows and 5 columns. The number of output neurons (20) was chosen after testing quantization and topographic errors (see [4]). At the end of the training, each year is set in a hexagon of the SOM map. Years appearing distant in the modeling space (according to the variables used during the training) represent expected climatic differences for real environmental characteristics. A k-means algorithm was applied to cluster the trained map. The SOM units (hexagons) were divided into clusters according to the weight vectors of the neurons, and clusters were justified according to the lowest Davis Bouldin Index (i.e., for a solution with low variance within clusters and high variance between clusters [3]). To analyze the contribution of climatic variables in clustering structures of the trained SOM, each input variable calculated during the training process was displayed in each neuron of the trained SOM in grey scale. This method clearly shows the discriminatory powers of input variables in mapping [1].

Second, we aimed to bring out the relationships between years, climatic variables, and biological variables concerning social wasps, the latter summarized by the number of wasp nests having successfully survived the entire (i.e., both the major and the short) rainy season each year in the same location. During the above-mentioned training, we used a mask function to assign a null weight to the two biological variables (number of wasp nests at KP 23, all species pooled, and percentage of *Polybia bistriata* nests); whereas the 12 climatic variables were assigned a weight of ‘1’ so that the ordination process was based on these biological variables only. Setting the mask value to zero for a given component removes the effect of that component on the organization of the map [5,6]. The values for the number of wasp nests and the percentages of *P. bistriata* nests were thus displayed on the SOM previously trained with climatic data only. This permitted a co-variation between these climatic data (that characterized the SOM) and biological variables characterizing the social wasps to be illustrated.

**References**

[1] Kohonen T (2001) Self-Organizing Maps, 3^rd^ ed. Springer, Berlin. 501p.

[2] Park YS, Céréghino R, Compin A, Lek S (2003) Applications of artificial neural networks for patterning and predicting aquatic insect species richness in running waters. Ecol Model 160: 265-280.

[3] Céréghino R, Park YS (2009) Review of the self-organizing map (SOM) approach in water resources: commentary. Environ Model Software 24: 945-947.

[4] Céréghino R, Park YS, Compin A, Lek S (2003) Predicting the species richness of aquatic insects in streams using a limited number of environmental variables. J North Amer Benthol Soc 22: 442-456.

[5] Sirola M, Lampi G, Parviainen J (2004) Using self-organizing map in a computerized decision support system. In: Pal N, Kasabov N, Mudi R, Pal S, Parui S, eds. Neural information processing. Berlin: Springer-Verlag. pp. 136-141

[6] Raivio K (2006) Analysis of soft handover measurements in 3G network. Proceedings of the 9th ACM international Symposium on Modeling Analysis and Simulation of Wireless and Mobile Systems (Terromolinos, Spain, October 02-06, 2006). New York: ACM Press. pp. 330-337
