# Supplementary material for: Climate Change Impact on Neotropical Social Wasps
Source: PLoS One. 2011 Nov 2;6(11):e27004. doi: 10.1371/journal.pone.0027004 (PMC3206903; doi:10.1371/journal.pone.0027004)
Supplement: Figure S1 — Variations in the number of wasp nests at the onset and after the 1998-2000 La Niña event. (DOC) [file pone.0027004.s003.doc]

**Supplementary Figure S1**. **Variations in the number of wasp nests (all species pooled) at the onset and after the 1998-2000 La Niña event**. The surveys were conducted at six sites along the road leading to Petit Saut in the Sinnamary district. Statistical comparisons (i.e., GLIM; multivariate repeated measures analysis) for the three years studied (F^2^_12_ = 76.43; P<0.001) permitting us to make pairwise comparisons showing that the three groups of years were significantly different from each other; 1998 *vs*. 2002 or 2006: P<0.001; 2002 *vs*. 2006 P<0.004.
