# Supplementary material for: Climate Change Impact on Neotropical Social Wasps
Source: PLoS One. 2011 Nov 2;6(11):e27004. doi: 10.1371/journal.pone.0027004 (PMC3206903; doi:10.1371/journal.pone.0027004)
Supplement: Figure S2 — Pluviometry in the area studied during the dry season, the short and the major rainy seasons between 1980 and 2009. (DOC) [file pone.0027004.s004.doc]

**Supplementary Figure S2**. **Pluviometry in the area studied during the dry season, the short and the major rainy seasons between 1980 and 2009**, with special reference to the contrast between the dry and the rainy seasons (difference between mean monthly rainfall). The tendency curves show a decrease in rainfall during the dry seasons and an increase during the short rainy seasons (greater contrast), while rainfall during the major rainy seasons was stable.
