# Supplementary material for: Climate Change Impact on Neotropical Social Wasps
Source: PLoS One. 2011 Nov 2;6(11):e27004. doi: 10.1371/journal.pone.0027004 (PMC3206903; doi:10.1371/journal.pone.0027004)
Supplement: Table S1 — Wasp species sampled during the surveys. (DOC) [file pone.0027004.s005.doc]

**Supplementary Table S1**. **Wasp species sampled during the surveys.** These surveys were conducted at the onset and after the 1997-2000 ENSO event along ca. 5 km of forest edges around Petit Saut. Statistical comparisons. Number of wasp nests: Kruskal-Wallis test H^3^_180_ = 71.43; P<0.0001; Dunn's multiple comparison test: 1997 *vs.* 2002 and 1997 *vs*. 2007: P<0.001; 2002 *vs*. 2007: NS.

*: 67 out of 119 *Polybia bistriata* nests (56.3%) sheltering under a large leaf of *Clusia grandiflora*.

| **Genus species subspecies author** | **Baseline survey 1997** | **November 2002 survey** | **November 2007 survey** |
| --- | --- | --- | --- |
| **Polistini** |  |  |  |
| *Polistes canadensis canadensis* (Linnaeus) | 31 | 3 | 1 |
| *Polistes geminatus* Fox | 4 | 2 | 1 |
| *Polistes occipitalis* Ducke | 1 | 0 | 0 |
| *Polistes pacificus* Fabricius | 19 | 9 | 5 |
| *Polistes testaceicolor* Bequaert | 3 | 1 | 1 |
| *Polistes versicolor versicolor* (Olivier) | 9 | 4 | 3 |
| **Mischocyttarini** |  |  |  |
| *Mischocyttarus collarellus* Richards | 3 | 0 | 0 |
| *Mischocyttarus injucundus* (de Saussure) | 11 | 6 | 3 |
| *Mischocyttarus lecointei* (Ducke) | 5 | 0 | 0 |
| *Mischocyttarus lemoulti* (du Buysson) | 1 | 0 | 0 |
| *Mischocyttarus prominulus* Richards | 2 | 0 | 0 |
| *Mischocyttarus punctatus* (Ducke) | 2 | 0 | 0 |
| *Mischocyttarus surinamensis* (de Saussure) | 3 | 0 | 0 |
| **Epiponini** |  |  |  |
| *Agelaia angulata* (Fabricius) | 1 | 0 | 0 |
| *Agelaia cajennensis* (Fabricius) | 1 | 0 | 0 |
| *Agelaia constructor* (de Saussure) | 1 | 0 | 0 |
| *Agelaia fulvofasciata* (DeGeer) | 3 | 0 | 0 |
| *Agelaia pallipes pallipes* (Olivier) | 1 | 0 | 0 |
| *Agelaia timida* Cooper | 1 | 0 | 0 |
| *Angiopolybia obidensis* (Ducke) | 2 | 0 | 0 |
| *Angiopolybia pallens* (Lepeletier) | 10 | 1 | 1 |
| *Apoica arborea* (Christ) | 0 | 2 | 2 |
| *Apoica pallens* (Fabricius) | 8 | 3 | 1 |
| *Apoica pallida* (Olivier) | 1 | 0 | 0 |
| *Apoica thoracica* du Buysson | 2 | 0 | 0 |
| *Brachygastra myersi* Bequaert | 9 | 0 | 0 |
| *Brachygastra lecheguana* (Latreille) | 1 | 0 | 0 |
| *Brachygastra scutellaris* (Fabricius) | 9 | 0 | 2 |
| *Brachygastra smithii* (de Saussure) | 24 | 0 | 3 |
| *Chartergellus amazonicus* Richards | 1 | 1 | 0 |
| *Charterginus xanthura* (de Saussure) | 5 | 0 | 0 |
| *Chartergus artifex* (de Saussure) | 0 | 1 | 1 |
| *Epipona tatua* (Cuvier) | 0 | 0 | 1 |
| *Metapolybia cingulata* (Fabricius) | 1 | 0 | 0 |
| *Metapolybia docilis* Richards | 1 | 0 | 0 |
| *Metapolybia nigra* Richards | 1 | 0 | 0 |
| *Parachartergus fraternus* (Gribodo) | 1 | 0 | 0 |
| *Parachartergus griseus* (Fox) | 2 | 0 | 0 |
| *Parachartergus smithii* (de Saussure) | 1 | 0 | 0 |
| *Polybia affinis* du Buysson | 1 | 0 | 0 |
| *Polybia belemensis belemensis* Richards | 4 | 0 | 0 |
| *Polybia bistriata* (Fabricius) * | 119 | 31 | 27 |
| *Polybia dimidiata* (Fabricius) | 1 | 0 | 0 |
| *Polybia emaciata* Lucas | 3 | 0 | 0 |
| *Polybia jurinei* de Saussure | 2 | 0 | 0 |
| *Polybia micans* Ducke | 8 | 0 | 0 |
| *Polybia occidentalis* (Olivier) | 5 | 0 | 0 |
| *Polybia platycephala platycephala* Richards | 4 | 0 | 0 |
| *Polybia platycephala sylvestris* Richards | 1 | 0 | 0 |
| *Polybia quadricincta* de Saussure | 2 | 0 | 0 |
| *Polybia rejecta* (Fabricius) | 19 | 9 | 9 |
| *Polybia scrobalis surinama* Richards | 33 | 10 | 5 |
| *Polybia striata* (Fabricius) | 1 | 0 | 0 |
| *Protopolybia duckei* (du Buysson) | 4 | 2 | 0 |
| *Protopolybia emortualis* (de Saussure) | 18 | 12 | 1 |
| *Protopolybia exigua binominata* Schulz | 1 | 0 | 0 |
| *Protopolybia minutissima* (Spinola) | 2 | 0 | 0 |
| *Protopolybia nitida* Ducke | 3 | 0 | 0 |
| *Protopolybia pallidibalteatus* (Cameron) | 2 | 0 | 0 |
| *Protopolybia rugulosa* Ducke | 1 | 0 | 0 |
| *Pseudopolybia compressa* (de Saussure) | 2 | 0 | 0 |
| *Pseudopolybia langi* Bequaert | 1 | 0 | 0 |
| *Pseudopolybia vespiceps* (de Saussure) | 4 | 0 | 0 |
| *Synoeca surinama* (Linnaeus) | 2 | 0 | 0 |
| Total number of wasp nests | 424 | 97 | 67 |
| Number of wasp species | 61 | 16 | 17 |
